# Genetic Susceptibility and Predictors of Paradoxical Reactions in Buruli Ulcer

**DOI:** 10.1371/journal.pntd.0004594

**Published:** 2016-04-20

**Authors:** Yves Thierry Barogui, Sandor-Adrian Klis, Roch Christian Johnson, Richard O. Phillips, Eveline van der Veer, Cleo van Diemen, Tjip S. van der Werf, Ymkje Stienstra

**Affiliations:** 1 Centre de Dépistage et de Traitement de l’Ulcère de Buruli de Lalo, Ministère de la Santé, Cotonou, Bénin; 2 Department of Internal Medicine, Infectious Diseases Service, University Medical Center Groningen, University of Groningen, Groningen, The Netherlands; 3 Centre Interfacultaire de Formation et de Recherche en Environnement pour le Développement Durable, Université d’Abomey-Calavi, Abomey-Calavi, Bénin; 4 Kwame Nkrumah University of Science and Technology, Kumasi, Ghana; 5 Department of Laboratory Medicine, University of Groningen, University Medical Center Groningen, Groningen, The Netherlands; 6 Department of Genetics, University of Groningen, University Medical Centre Groningen, Groningen, The Netherlands; 7 Department of Pulmonary Diseases & Tuberculosis, University of Groningen, University Medical Centre Groningen, Groningen, The Netherlands; University of Tennessee, UNITED STATES

## Abstract

**Introduction:**

Buruli ulcer (BU) is the third most frequent mycobacterial disease in immunocompetent persons after tuberculosis and leprosy. During the last decade, eight weeks of antimicrobial treatment has become the standard of care. This treatment may be accompanied by transient clinical deterioration, known as paradoxical reaction. We investigate the incidence and the risks factors associated with paradoxical reaction in BU.

**Methods:**

The lesion size of participants was assessed by careful palpation and recorded by serial acetate sheet tracings. For every time point, surface area was compared with the previous assessment. All patients received antimicrobial treatment for 8 weeks. Serum concentration of 25-hydroxyvitamin D, the primary indicator of vitamin D status, was determined in duplex for blood samples at baseline by a radioimmunoassay. We genotyped four polymorphisms in the *SLC11A1* gene, previously associated with susceptibility to BU. For testing the association of genetic variants with paradoxical responses, we used a binary logistic regression analysis with the occurrence of a paradoxical response as the dependent variable.

**Results:**

Paradoxical reaction occurred in 22% of the patients; the reaction was significantly associated with trunk localization (p = .039 by Χ^2^), larger lesions (p = .021 by Χ^2^) and genetic factors. The polymorphisms 3’UTR TGTG ins/ins (OR 7.19, p < .001) had a higher risk for developing paradoxical reaction compared to ins/del or del/del polymorphisms.

**Conclusions:**

Paradoxical reactions are common in BU. They are associated with trunk localization, larger lesions and polymorphisms in the *SLC11A1* gene.

## Author Summary

Buruli ulcer is an infectious disease of skin, subcutaneous fat and sometimes bone, mainly affecting children in West Africa. It is considered as one of the Neglected Tropical Diseases but the disease occurs also in moderate climates like South East Australia and Japan where it may also affect adults. Once a patient has started antibiotic treatment, lesions may increase in size even if the antimicrobial treatment is effective; this is highly confusing for doctors and patients as they may think that treatment actually fails. The cause of Buruli ulcer is *Mycobacterium ulcerans*, related to other mycobacteria that cause disease in man, like leprosy and tuberculosis. Using data from two different studies in West Africa, we show that these paradoxical reactions are associated with trunk localization and that they occur more often in larger lesions. The chance to develop these reactions appeared partly inherited: carrying the homozygous ins/ins genotype of 3’UTR TGTG 285 polymorphism in the SLC11A1 gene increased the risk of paradoxical reactions. Vitamin D is important for the immune defense against infections by mycobacteria. Vitamin D blood concentrations were not associated with paradoxical reactions; patients generally did well, and we did not need corticosteroid immune suppression to overcome these reactions.

## Introduction

The neglected tropical disease Buruli ulcer (BU) is the third most frequent mycobacterial disease in immunocompetent persons after tuberculosis and leprosy [1–2]. It is caused by *Mycobacterium ulcerans*. Central to the pathogenesis is the immunosuppressant and necrosis inducing toxin mycolactone.

During the last decade, an antibiotic regimen of eight weeks of streptomycin and rifampicin was introduced [3,4]. Earlier studies reported the success of this antimicrobial treatment with or without surgery [5–7]. A clinical trial showed that antimicrobial treatment was highly effective in patients with small lesions (cross-sectional diameter < 10 cm), of which 96% healed without surgery [8].

However, during or after antibiotic treatment the BU lesions may worsen. This could be caused by treatment failure [9–11], but might also be due to an inflammatory response caused by treatment-induced recovery of the immune system, i.e. a paradoxical reaction. Paradoxical reactions have been described in tuberculosis and in leprosy [12,13]. Recent studies have recognized the existence of paradoxical reactions in BU [11,14]. In Australia, one in five BU patients appear to have a paradoxical reaction. Most cases occurred between three and ten weeks after the start of treatment [9]. In a trial in Ghana, most of the cases with a paradoxical reaction (>30%) were reported at week eight after the beginning of antimicrobial treatment [15]. The diagnosis of paradoxical response is difficult; no serological markers have been identified to differentiate paradoxical reactions from treatment failure [15]. Paradoxical reactions can be defined clinically by worsening of existing lesions, or the appearance of new lesions, and histologically by the appearance of intense inflammation in lesions [9]. Importantly, in most areas endemic for BU, histology is not available. In Africa, very few studies have addressed paradoxical reactions in BU [10,14] as well as its risk factors. In Australia, edematous lesions, use of amikacin and age above sixty years old were strongly associated with paradoxical reactions. In addition to sociodemographic and clinical features, we suggest genetic factors may influence the occurrence of paradoxical reactions as well. As paradoxical reactions are hypothesized to reflect an exaggerated immune response, genes involved in the immune response in infectious diseases might play a role. For BU, a polymorphism in the innate immune *SLC11A1* gene (formerly known as *NRAMP1*) was previously found to be associated with increased susceptibility to BU [16]. Furthermore it has been shown that 1,25(OH)_2_D_3_ suppresses the Th1 response by down-regulating the production of pro-inflammatory cytokines [17–19]. So it is possible that polymorphisms in *SLC11A1* gene as well as vitamin D are also related to paradoxical reactions.

In West Africa, most of the patients are below age 15 [20] and amikacin is not used to treat BU but very few patients receive antimicrobial treatment without streptomycin, the parent aminoglycoside drug. As the patient demographics and treatment regimen in West-Africa are widely different from that of Australia, it is important to look at the risk factors for developing paradoxical reactions in BU in this region. In Ghana, paradoxical reactions were described in patients with *M*. *ulcerans* infection with early lesions (duration < 6 months), limited to 10 cm cross-sectional diameter [14]; large lesions that are common in west Africa were not included in that study. Our study focuses on the risk factors associated with paradoxical reactions in patients with both small and large BU lesions, during and after antimicrobial treatment, and examines the influence of genetic factors as well.

## Methods

### Study population

In the present study, we included participants of two randomized clinical trials in Ghana and Benin. The BURULICO drug trial with patients enrolled between 2006–2008, was a randomized controlled trial for the treatment of early (duration less than 6 months), limited (cross-sectional diameter, 10 cm) *M*. *ulcerans* infection [clintrials NCT00321178]. In this trial, patients were randomized to receive either 8 weeks of streptomycin and rifampicin or 4 weeks of streptomycin and rifampicin followed by 4 weeks of clarithromycin and rifampicin. Participants in this study that had their BU lesions healed at time point 52 weeks after initiation of antimicrobial treatment were earlier studied for possible paradoxical reactions [14]. The second trial is a randomized trial on timing of the decision on surgical intervention for BU patients treated with rifampicin and streptomycin [clintrials NCT01432925]. All included patients (2011–2015) had confirmed *M*. *ulcerans* infection by direct microscopy following acid-fast staining or Polymerase Chain Reaction (PCR), and all received 8 weeks of antimicrobial therapy with rifampicin and streptomycin. For both trials, patients who were pregnant, children below five years old, patients not compliant with the antibiotic therapy, and patients with osteomyelitis, were excluded from the study. For the current study population, 150 of 241 participants of the BURULICO study, and 91 of the Burulitime study contributed (S1 Dataset).

### Study design

For all patients, we recorded demographics and clinical data from the trial databases. In addition, we recorded the progression of the size of the lesion size by measurement at regular intervals. For both trials, measurements were available for the first 12 weeks at two-week intervals. In the BURULICO trial, lesions were measured at 14, 21, 27 weeks after start of treatment, and for the timing of surgical intervention trial, measurements were available at 16, 20, and 28 weeks after starting treatment. For analyses, the measurements at 14 and 16 weeks, at 21 and 20 weeks, and 27 and 28 weeks were considered to be equivalent time points.

### Method of measurement

#### Lesion size

Lesion measurement included the indurated area around the visible skin defect; sloughing of indurated skin is an expected clinical course and does not per se constitute a paradoxical reaction. This lesion measurement was drawn on an acetate sheet and the surface area was calculated for every individual lesion at the different time point of the follow up.

#### Vitamin D serum concentration

Baseline vitamin D serum concentrations were available for patients participating in the BURULICO trial only. Blood samples in clotted blood tubes for serum were cooled until centrifuged within 24 hours after collection, then stored at -20°C, and sent in frozen condition from Ghana to the University Medical Center Groningen, the Netherlands, until processed. Serum concentration of 25-hydroxyvitamin D, the primary indicator of vitamin D status, was determined in duplex for every sample by a radioimmunoassay (DiaSorin, Stillwater, MN) [21]. The mean of these two results was used for the analyses. Currently, there is no international consensus on the optimal level for vitamin D [22]. In healthy humans, vitamin D adequacy is defined as the presence of 25(OH)D_3_ at a concentration of 50–75 nmol/L, levels of 75 nmol/L or greater represent vitamin D sufficiency while the serum levels of 25–50 nmol/L of circulating 25(OH)D_3_ are defined as vitamin D insufficiency [23,24].

#### Genetic assays

Genetic data were obtained from patients participating in the BURULICO trial. Participants in the trial in Benin on timing on surgical intervention, were not asked for consent to testing of genetic susceptibility and could therefore not be included. The *SLC11A1* gene has been previously associated with susceptibility to BU [16]. We genotyped four polymorphisms in the *SLC11A1* gene: rs59823161 (3’UTR TGTG ins/del); rs17235409 (D543 G/A); rs3731865 (INT4 G/C); and a (CA)n microsatellite in the immediate 5’ region of the gene, as described previously [16]. Normal genotype was designated as having a microsatellite length of 200 base pairs and variant genotypes, having microsatellite lengths of 202 or 204 were pooled as “other”.

### Definition of paradoxical response

We considered an increase in lesion area of more than 5% between two consecutive measurements as a clinically relevant change. We defined a paradoxical reaction as 2 consecutive increases in lesion size after 1 initial decrease. We additionally performed all analyses (post-hoc) using a less strict definition of two consecutive increases without an initial decrease.

### Statistics

For associations of clinical and patient characteristics with paradoxical responses, we used t-tests or Mann-Whitney U tests for accordingly and Χ^2^ tests for categorical variables. For testing the association of genetic mutations and variants with paradoxical responses, we used a binary logistic regression analysis with the occurrence of a paradoxical response as the dependent variable.

### Ethics

The protocol was approved by the Committee on Human Research, Publication, and Ethics of the Kwame Nkrumah University of Science and Technology and the Komfo Anokye Teaching Hospital, Kumasi (CHRPE/07/01/05), by the Ethical Review Committee of Ghana Health Services (GHS-ERC-01/01/06) and by the provisional national ethical review board of the Ministry of Health Benin, nr IRB00006860. Written and verbal informed consent or assent was obtained from all participants aged ≥12 years, and consent from parents, caretakers, or legal representatives of participants aged ≤18 years. All data were analyzed anonymously.

## Results

### Patient and clinical characteristics

A total of 241 patients were included, 150 from Ghana, and 91 from Benin; 61% were female. The mean (SD) age was 16.2 (13.2) years. On presentation, 45% of patients had an ulcer, 23% had a plaque, and 13% had a nodule; 29% had a WHO category I lesion, 55% a category II lesion, and 16% a category III lesion. The median (IQR) surface area of the lesion on presentation was 20.6 (6.6; 43.5) cm^2^; 49% had a lesion on the lower limb, 43% on the upper limb, and 8% on the trunk.

### Paradoxical reactions

**Fig 1:**
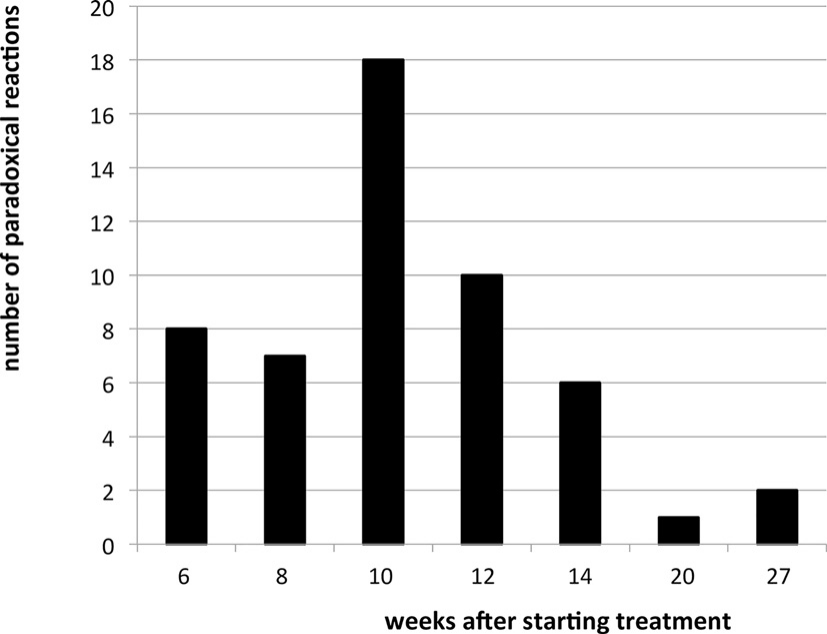
Number of paradoxical reactions by weeks after starting treatment.

Paradoxical reactions, as defined by an initial decrease of the lesion followed by two consecutive increases occurred in 22% of cases. Most paradoxical reactions occurred between weeks 8 and 12 (Fig 1). When using a definition that did only require two consecutive increases without an initial decrease, 26% of patients had a paradoxical response, and the frequency distribution of the initiation week of paradoxical reaction did not differ substantially. All cases that had a paradoxical reaction healed without additional treatment.

### Associations with patient and lesion characteristics

Paradoxical reactions were significantly related to the site of lesion (*p =* .039 by Χ^2^): 44% of patients with a lesion on the trunk had a paradoxical response, compared to 24% of patients with a lesion on the upper limb, and 17% with a lesion on the lower limb.

Paradoxical reactions were also significantly related to WHO category at presentation. Ten percent of patients with a category I lesion had a paradoxical response, compared to 27%, and 23% of patients with a category II and category III lesion, respectively (*p* = .021 by Χ^2^). Paradoxical reactions were not significantly related to patient age or gender. They were also not related to the type of lesion, duration of lesion before presentation, or white blood cell count at presentation (Table 1). For the participants in the BURULICO trial, paradoxical reactions were not related to treatment arm (8 week streptomycin vs 4 weeks streptomycin followed by 4 weeks clarithromycin). The pulse and temperature at the time of paradoxical response did not differ from the pulse at presentation by paired samples t-test, and did not differ from the average pulse and temperature of those not classified as having a paradoxical response at the respective week. Using the less strict definition, the same pattern of results emerged, where paradoxical reactions were significantly related to the site of the lesion (p = .024 by Χ^2^) and WHO category at presentation (p = .009 by Χ^2^), but to none of the other variables.

**Table 1:** Risk factors associated with paradoxical reactions.

Risk factor		PR	No PR	OR (95%CI)	p-value
**Age mean (SD)**		15.2 (12.5)	16.5 (13.4)	N/A	0.529*
**Gender n (%)**	Female	36 (24%)	117 (76%)	1	
	Male	16 (18%)	72 (88%)	0.72 (0.37–1.40)	0.416ǂ
**WHO categoryn (%)**	CAT 1	7 (10%)	62 (90%)	1	
	CAT 2	36 (27%)	97 (73%)	3.29 (1.38–7.87)	0.007ǂ
	CAT 3	9 (23%)	30 (77%)	2.66 (0.90–7.81)	0.076ǂ
**site of lesion n (%)**	Lower limb	20 (17%)	97 (83%)	1	
	Upper limb	24 (24%)	76 (76%)	1.54 (0.79–2.94)	0.209ǂ
	Trunk	8 (44%)	10 (56%)	3.88 (1.36–11.1)	0.011ǂ
**Type of lesion n (%)**	Ulcerative	29 (20%)	113 (80%)	1	
	Non-ulcerative	23 (23%)	76 (76%)	1.18 (.64–2.19)	0.602ǂ
**Vitamin D nmol/Lmean (SD)**		66.5 (19.1)	68.3 (17.1)	N/A	0.631*
**3’UTR TGTG n (%)**	ins/ins	22 (31%)	48 (69%)	1	
	ins/del	4 (6%)	63 (94%)	0.14 (0.05–0.44)	0.001ǂ
	del/del	1 (13%)	7 (87%)	0.44 (0.04–4.55)	0.494ǂ
**D543N n (%)**	G/G	25 (21%)	96 (79%)	1	
	G/A	2 (8%)	23 (92%)	3.00 (0.66–13.51)	0.155ǂ
	A/A	0 (0%)	0 (0%)	N/A	
**INT4 n (%)**	G/G	21 (17%)	105 (83%)	1	
	G/C	5 (28%)	13 (72%)	1.56 (.55–4.43)	0.405ǂ
	C/C	1 (100%)	0 (0%)	N/A	
**5’(CA)**_**n**_ **n (%)**	200/200	9 (14%)	60 (86%)	1	
	200/other	17 (25%)	52 (75%)	2.18 (.90–5.30)	0.086ǂ
	other/other	1 (13%)	7 (87%)	0.98 (.32–2.95)	0.975ǂ

* = t-test

ǂ = Binary logistic regression, PR = paradoxical reaction, OR = Odds ratio, CI = confidence interval.

### Associations with vitamin D and polymorphisms in the SLC11A1 gene

Vitamin D deficiency was found in 15% of participants. The mean (SD) vitamin D level was 66.5 (19.1) for the patients who had paradoxical reaction and 68.3 (17.1) for those who did not; 38% of patients with a vitamin D deficiency had a paradoxical reaction, compared to 23% of patients without a deficiency (*p =* .134 by Χ^2^). In the post-hoc analysis using the less strict definition of a paradoxical response, 33% of patients with a vitamin D deficiency had a paradoxical reaction, compared to 17% of patients without a deficiency (*p =* .082 by Χ^2^).

31% of patients with a 3’UTR TGTG ins/ins polymorphism had a paradoxical response, compared to 13% of patients with a ins/del or del/del polymorphism (OR 0.14, 95% CI: 0.05–0.44). 5’(CA)_n_ microsatellite length, INT4 G/C polymorphism and D543N G/G polymorphism were not significantly related to paradoxical responses (Table 1). Using the less strict definition of a paradoxical response in a post-hoc analysis, a similar pattern of results emerged.

## Discussion

This is the first prospective study in West Africa addressing risk factors associated with paradoxical reaction in BU. In our sample, paradoxical reactions were common, and significantly associated with trunk localization, larger lesions and genetic factors. Currently, there is no standard definition of paradoxical reactions in BU. Histological aspects [9] suggested from Australia is not feasible in rural West Africa where most BU cases occur [2]. All patients included in this study healed without changes in therapy (no change in antibiotics, no corticosteroids). This strongly supports our suggested definition and suggests that cases in our study represent true paradoxical reaction and not progressive disease secondary to antibiotic failure.

We found a 22% incidence of paradoxical reactions (2 consecutive increases after 1 initial decrease and healing without surgery or a change in antimicrobial therapy), which is similar to a previous study from Australia [9].

In our study, most paradoxical reactions occurred between week 8 and 12—slightly later than the Australian study, where most paradoxical reactions occurred between week 3 and 10 [9,11]. In the case reports from Benin paradoxical reactions occurred between 12 and 409 days after completion of antibiotic treatment [10].

Mycolactone, the exotoxin produced and secreted by *M*. *ulcerans*, has been proposed as the major cause of immune suppression [28–32]. Perhaps, the period between week 8 and 12 in which most paradoxical reactions occurred coincides with the elimination of most *M*. *ulcerans* organisms, with an arrest in the production and subsequently, a strong decrease in tissue concentration of mycolactone. The increase of the lesion then reflects an inflammatory response against the microbes—or microbial antigens of dead bacilli—already present in tissue which initially failed to elicit a host immune response [25–27,30].

We found several risk factors associated with paradoxical reactions. The incidence appeared to increase in larger lesions. One explanation of this may be that smaller lesions heal before eight weeks when most of the paradoxical reaction occurs. Another possibility is that larger lesions have a higher bacterial load than small lesions. We showed that lesions localized on trunk were significantly associated with paradoxical reaction, even when controlling for the size of the lesion. More than 4 out 10 patients (44%) with lesion on the trunk had paradoxical reaction compared to 24% and 17% for the upper limb and lower limb respectively. The increased incidence of paradoxical reactions on the trunk might be due to a difference in local immune responses and body temperature.

Our study shows that paradoxical reactions were not significantly associated with patient age or type of lesion. This finding contrasts with Australian patients in whom associations between paradoxical reactions and age and edema were reported [9]. This might be due to differences in the study populations. In affluent countries like Australia, with a steeper population pyramid, BU mainly affects the elderly in Australia [31], while in West Africa, most patients are children [32].

Paradoxical reactions were not associated with the white blood cell count or patients’ vital parameters such as the temperature and the pulse rate. We argue that an increase of pulse, temperature or white blood cell count is indicative of an additional disease or super-infection, which should be further investigated. Whether paradoxical reactions were associated with aminoglycoside use, as has been shown for amikacin in Australia, could not be examined for streptomycin use because all study participants had been exposed to this drug, for 4 or 8 weeks. One might speculate that this effect seen in amikacin might in fact reflect a decrease in paradoxical reactions by using antimicrobial drugs like macrolides that have been associated with immuno-modulatory effects [33].

We also show for the first time that paradoxical reactions to *M*. *ulcerans* infection are associated with genetic factors. Carrying the homozygous ins/ins genotype of 3’UTR TGTG polymorphism in the *SLC11A1* increases the risk of paradoxical reactions in BU. Earlier studies have shown that genetic factors can influence the innate immune response to mycobacterial antigens, such as infectious disease susceptibility genes, e.g., *SLC11A1*, HLA-DR, vitamin D_3_ receptor, and mannose binding protein [34,35]. In BU no associations were found with the 3’UTR TGTG ins/del polymorphism and developing BU [16]. However in tuberculosis, it was reported that participants who were heterozygous for two *SLC11A1* polymorphisms (*INT4* and 3’UTR) were at highest risk of tuberculosis [35]. A meta-analysis [35] has shown that the TGTG ins/ins 3’UTR genotype protected against tuberculosis, compared to the del/del genotype. We interpret our data such that the protective TGTG ins/ins 3’UTR genotype in the *SLC11A1* gene may induce a stronger immune response during *M*. *ulcerans* infection. In turn, this stronger immune response might increase the risk of paradoxical reactions once BU develops. It has been reported that genetic variation in *SLC11A1* affects susceptibility to others mycobacterial diseases such as leprosy and tuberculosis [35–37]. However, no study addressed the genetic risk factor for paradoxical reaction in tuberculosis or leprosy.

In this study, we report for the first time that paradoxical reactions are not associated with vitamin D level. Vitamin D deficiency has been found to be associated with susceptibility to tuberculosis [38]. Very few studies address vitamin D and paradoxical reactions in tuberculosis. Clearing of pathogens with anti-tuberculosis treatment and a delayed negative feedback on macrophage activation due to low 1,25(OH)_2_D production from vitamin D deficiency can lead to excessive granuloma formation and an exacerbated inflammatory response [39]. In our sample, the means of vitamin D level in patients with or without paradoxical reactions were similar.

All included patients in this study healed without any change in therapy. In earlier studies corticosteroids were used to treat paradoxical reactions [9,40,41]. We would indeed caution for use of corticosteroids West Africa, as other infections like tuberculosis and strongyloidiasis may worsen.

This study has some limitations. There are no standard definitions of paradoxical reactions in BU that we could use to validate our definition. Our definition is clinical and did not include histological aspects, which may lead to a lack of accuracy. However we believe that our cases accurately represent paradoxical reactions since all patients healed without any additional therapy. Secondly, we excluded co-infected patients with Buruli ulcer and HIV. This may have reduced the incidence and severity [42].

Paradoxical reactions are common in BU–and it is important that these should be differentiated from antimicrobial treatment failure. These paradoxical reactions are associated with trunk localization, larger lesions and certain polymorphisms in the *SLC11A1* gene. There was no apparent need to change therapy or add steroids.

## Supporting Information

S1 Dataset Supplemental data. (SAV)

## References

[1] van der Werf TS, van der Graaf WT, Tappero JW, Asiedu K (1999) *Mycobacterium ulcerans* infection. Lancet 354: 1013–1018. 10501380 10.1016/S0140-6736(99)01156-3

[2] van der Werf TS, Stienstra Y, Johnson RC, et al (2005) *Mycobacterium ulcerans* disease. Bull World Health Organ 83: 785–791. 16283056 PMC2626418

[3] Johnson PD, Hayman JA, Quek TY, et al (2007) Consensus recommendations for the diagnosis, treatment and control of *Mycobacterium ulcerans* infection (Bairnsdale or Buruli ulcer) in Victoria, Australia. Med J Aust 186: 64–68. 17223765 10.5694/j.1326-5377.2007.tb00802.x

[4] World Health Organisation (2012) Treatment of *Mycobacterium ulcerans* disease(Buruli ulcer): guidance for health workers. Geneva, Switzerland.

[5] Chauty A, Ardant MF, Adeye A, et al (2007) Promising clinical efficacy of streptomycin-rifampin combination for treatment of buruli ulcer (*Mycobacterium ulcerans* disease). Antimicrob Agents Chemother 51: 4029–4035. 17526760 10.1128/AAC.00175-07 PMC2151409

[6] Sarfo FS, Phillips R, Asiedu K, et al (2010) Clinical efficacy of combination of rifampin and streptomycin for treatment of *Mycobacterium ulcerans* disease. Antimicrob Agents Chemother 54: 3678–3685. 10.1128/AAC.00299-10 20566765 PMC2935024

[7] Kibadi K, Boelaert M, Fraga AG, et al (2010) Response to treatment in a prospective cohort of patients with large ulcerated lesions suspected to be Buruli Ulcer (*Mycobacterium ulcerans* disease). PLoS Negl Trop Dis 4: e736 10.1371/journal.pntd.0000736 20625556 PMC2897843

[8] Nienhuis WA, Stienstra Y, Thompson WA, Awuah PC, Abass KM, et al (2010) Antimicrobial treatment for early, limited mycobacterium ulcerans infection: A randomised controlled trial. Lancet 375: 664–672. 10.1016/S0140-6736(09)61962-0 20137805

[9] O'Brien DP, Robson M, Friedman ND, et al (2013) Incidence, clinical spectrum, diagnostic features, treatment and predictors of paradoxical reactions during antibiotic treatment of *Mycobacterium ulcerans* infections. BMC Infect Dis 13: 416 10.1186/1471-2334-13-416 24007371 PMC3854792

[10] Ruf MT, Chauty A, Adeye A, et al (2011) Secondary Buruli ulcer skin lesions emerging several months after completion of chemotherapy: paradoxical reaction or evidence for immune protection? PLoS Negl Trop Dis 5: e1252 10.1371/journal.pntd.0001252 21829740 PMC3149035

[11] O'Brien DP, Robson ME, Callan PP, McDonald AH (2009) "Paradoxical" immune-mediated reactions to *Mycobacterium ulcerans* during antibiotic treatment: a result of treatment success, not failure. Med J Aust 191: 564–566. 19912091 10.5694/j.1326-5377.2009.tb03313.x

[12] Walker SL, Lockwood DN (2008) Leprosy type 1 (reversal) reactions and their management. Lepr Rev 79: 372–386. 19274984

[13] Hawkey CR, Yap T, Pereira J, et al (2005) Characterization and management of paradoxical upgrading reactions in HIV-uninfected patients with lymph node tuberculosis. Clin Infect Dis 40: 1368–1371. 15825042 10.1086/429317

[14] Nienhuis WA, Stienstra Y, Abass KM, et al (2012) Paradoxical responses after start of antimicrobial treatment in *Mycobacterium ulcerans* infection. Clin Infect Di*s* 54: 519–526. 10.1093/cid/cir856 22156855

[15] de Zeeuw J, Duggirala S, Nienhuis WA, et al (2013) Serum levels of neopterin during antimicrobial treatment for *Mycobacterium ulcerans* infection. Am J Trop Med Hyg 89: 498–500. 10.4269/ajtmh.12-0599 23836576 PMC3771288

[16] Stienstra Y, van der Werf TS, Oosterom E, et al (2006) Susceptibility to Buruli ulcer is associated with the SLC11A1 (NRAMP1) D543N polymorphism. Genes Immun 7: 185–189. 16395392 10.1038/sj.gene.6364281

[17] Lemire JM, Adams JS, Kermani-Arab V, et al (1985) 1,25-Dihydroxyvitamin D3 suppresses human T helper/inducer lymphocyte activity in vitro. J Immunol 134: 3032–3035. 3156926

[18] Lemire JM, Archer DC, Beck L, Spiegelberg HL (1995) Immunosuppressive actions of 1,25-dihydroxyvitamin D3: preferential inhibition of Th1 functions. J Nutr 125: 1704S–1708S. 7782931 10.1093/jn/125.suppl_6.1704S

[19] Selvaraj P, Harishankar M, Singh B, Banurekha VV, Jawahar MS (2012) Effect of vitamin D3 on chemokine expression in pulmonary tuberculosis. Cytokine 60: 212–219. 10.1016/j.cyto.2012.06.238 22800603

[20] Sopoh GE, Johnson RC, Chauty A, et al (2007) Buruli ulcer surveillance, Benin, 2003–2005. Emerg Infect Dis 13: 1374–1376. 10.3201/eid1309.061338 18252113 PMC2857274

[21] Hollis BW, Kamerud JQ, Selvaag SR, Lorenz JD, Napoli JL (1993) Determination of vitamin D status by radioimmunoassay with an ^125^I-labeled tracer. Clin Chem 39: 529–533. 8448871

[22] Selvaraj P, Harishankar M, Afsal K (2015) Vitamin D: Immuno-modulation and tuberculosis treatment. Can J Physiol Pharmacol 93: 377–384. 10.1139/cjpp-2014-0386 25744368

[23] Holick MF (2007) Vitamin D deficiency. N Engl J Med 357: 266–281. 17634462 10.1056/NEJMra070553

[24] Pearce SH, Cheetham TD (2010) Diagnosis and management of vitamin D deficiency. BMJ 340: b5664 10.1136/bmj.b5664 20064851

[25] Simmonds RE, Lali FV, Smallie T, Small PL, Foxwell BM (2009) Mycolactone inhibits monocyte cytokine production by a posttranscriptional mechanism. J Immunol 182: 2194–2202. 10.4049/jimmunol.0802294 19201873

[26] Torrado E, Fraga AG, Logarinho E, et al (2010) IFN-gamma-dependent activation of macrophages during experimental infections by *Mycobacterium ulcerans* is impaired by the toxin mycolactone. J Immunol 184: 947–955. 10.4049/jimmunol.0902717 20008288

[27] Boulkroun S, Guenin-Mace L, Thoulouze MI, et al (2010) Mycolactone suppresses T cell responsiveness by altering both early signaling and posttranslational events. J Immunol 184: 1436–1444. 10.4049/jimmunol.0902854 20042571

[28] van der Werf TS, Stinear T, Stienstra Y, van der Graaf WT, Small PL (2003) Mycolactones and *Mycobacterium ulcerans* disease. Lancet 362: 1062–1064. 14522538 10.1016/S0140-6736(03)14417-0

[29] Kishi Y. Chemistry of mycolactones, the causative toxins of Buruli ulcer (2011) Proc Natl Acad Sci USA 108: 6703–6708. 10.1073/pnas.1015252108 21383136 PMC3084064

[30] Phillips R, Sarfo FS, Guenin-Mace L, et al (2009) Immunosuppressive signature of cutaneous Mycobacterium ulcerans infection in the peripheral blood of patients with buruli ulcer disease. J Infect Dis 200: 1675–1684. 10.1086/646615 19863437

[31] Boyd SC, Athan E, Friedman ND, et al (2012) Epidemiology, clinical features and diagnosis of *Mycobacterium ulcerans* in an Australian population. Med J Aust 196: 341–344. 22432674 10.5694/mja12.10087

[32] Huang GK, Johnson PD. Epidemiology and management of Buruli ulcer (2014) Expert Rev Anti Infect Ther 12: 855–865. 10.1586/14787210.2014.910113 24918117

[33] Altenburg J, de Graaff CS, van der Werf TS, Boersma WG (2011) Immunomodulatory effects of macrolide antibiotics—part 1: biological mechanisms. Respiration 81(1):67–74. 10.1159/000320319 20733281

[34] Bellamy R (1998) Genetics and pulmonary medicine. 3. Genetic susceptibility to tuberculosis in human populations. Thorax 53: 588–593. 9797760 10.1136/thx.53.7.588 PMC1745258

[35] Bellamy R, Ruwende C, Corrah T, et al (1998) Variations in the NRAMP1 gene and susceptibility to tuberculosis in West Africans. N Engl J Med 338: 640–644. 9486992 10.1056/NEJM199803053381002

[36] Li X, Yang Y, Zhou F, Zhang Y, Lu H, et al (2011) SLC11A1 (NRAMP1) Polymorphisms and Tuberculosis Susceptibility: Updated Systematic Review and Meta-Analysis. PLoS ONE 6(1): e15831 10.1371/journal.pone.0015831 21283567 PMC3026788

[37] Abel L, Sanchez FO, Oberti J, Thuc NV, Hoa LV et al (1998) Susceptibility to leprosy is linked to the human NRAMP1 gene. J Infect Dis 177: 133–145. 9419180 10.1086/513830

[38] Wilkinson RJ, Llewelyn M, Toossi Z, et al (2000) Influence of vitamin D deficiency and vitamin D receptor polymorphisms on tuberculosis among Gujarati Asians in west London: a case-control study. Lancet 355: 618–621. 10696983 10.1016/S0140-6736(99)02301-6

[39] Conesa-Botella A, Mathieu C, Colebunders R, et al (2009). Is vitamin D deficiency involved in the immune reconstitution inflammatory syndrome? AIDS Res Ther 6: 4 10.1186/1742-6405-6-4 19383117 PMC2678152

[40] Friedman ND, McDonald AH, Robson ME, O'Brien DP (2012). Corticosteroid use for paradoxical reactions during antibiotic treatment for *Mycobacterium ulcerans*. PLoS Negl Trop Dis 6: e1767 10.1371/journal.pntd.0001767 23029568 PMC3459890

[41] Trevillyan JM, Johnson PD. Steroids control paradoxical worsening of *Mycobacterium ulcerans* infection following initiation of antibiotic therapy (2013) Med J Aust 198: 443–444. 23641997 10.5694/mja12.11559

[42] Wanda F, Nkemenang P, Ehounou G, et al (2014) Clinical features and management of a severe paradoxical reaction associated with combined treatment of Buruli ulcer and HIV co-infection. BMC Infect Dis 14: 423 10.1186/1471-2334-14-423 25073531 PMC4122778

